# Efficacy and safety of moxibustion in female infertility patients undergoing in vitro fertilization and embryo transfer: A systematic review protocol

**DOI:** 10.1097/MD.0000000000017560

**Published:** 2019-11-01

**Authors:** Tinghui Hou, Qianhua Zheng, Xiumei Feng, Ying Liu, Lu Wang, Ying Li

**Affiliations:** a Acupuncture and Moxibustion School; b Graduate School, Chengdu University of Traditional Chinese Medicine, Jinniu District, Chengdu, Sichuan, China.

**Keywords:** female infertility, in vitro fertilization and embryo transfer, moxibustion, protocol, systematic review

## Abstract

**Introduction:**

The purpose of this paper is to evaluate the efficacy and safety of moxibustion in infertility females/women undergoing in vitro fertilization and embryo transfer (IVF-ET).

**Methods and analysis:**

We will electronically search PubMed, Medline, Embase, Web of Science, the Cochrane Central Register of Controlled Trial, China National Knowledge Infrastructure, China Biomedical Literature Database, China Science Journal Database, and Wan-fang Database from their inception. Also, we will manually retrieve other resources, including reference lists of identified publications, conference articles, and grey literature. The clinical randomized controlled trials or quasi-randomized controlled trials related to moxibustion in female infertility patients undergoing IVF-ET will be included in the study. The language is limited to Chinese and English. Research selection, data extraction, and research quality assessment will be independently completed by 2 researchers. Data were synthesized by using a fixed effect model or random effect model depend on the heterogeneity test. The clinical total effective rate and the clinical pregnancy rate will be the primary outcomes. Ovulation rate, endometrial thickness, hormone level, traditional Chinese medicine (TCM) Syndrome Integral Scale and the adverse event will also be assessed as secondary outcomes. RevMan V.5.3 statistical software will be used for meta-analysis, and the level of evidence will be assessed by Grading of Recommendations Assessment, Development, and Evaluation (GRADE). Continuous data will be expressed in the form of weighted mean difference or standardized mean difference with 95% confidence intervals (CIs), while dichotomous data will be expressed in the form of relative risk with 95% CIs.

**Results:**

This study will provide a high-quality comprehensive evaluation of the efficacy and safety of moxibustion in the treatment of female infertility patients undergoing IVF-ET.

**Conclusion:**

This review will provide evidence to judge for judging whether moxibustion is effective in treating female infertility patients undergoing IVF-ET.

**Systematic review registration:**

PROSPERO, CRD42019135593

## 1 Introduction

Infertility refers to the cohabitation of adult men and women with a normal and regular sexual life history of >1 year, in the absence of any contraceptive measures and women failed to be pregnant.^[1,2]^ Its global incidence ranges from 9% to 18%.^[3]^ Studies have shown that there are 186 million people worldwide suffering from infertility, and the incidence varies from region to region, but it is high in developing countries.^[4,5]^ Infertility is often divided into primary and secondary types in clinic,^1^ and the secondary infertility is the most common female infertility in the world.^[6]^ It is a complex interaction of multiple factors, including functional, biological, and environmental impacts.^[7,8]^ The main causes of female infertility include reproductive system diseases, abnormal immune function, psychological factors, cultural level, and age of pregnancy.^[5,9,10]^ Among them, the reproductive system diseases such as the polycystic ovary syndrome (PCOS), tubal obstruction, tuberculosis, and other infections caused by pelvic inflammation, endometriosis, and so on are the main complaints from infertility females.^[11–13]^ In addition, up to 20% of infertile couples cannot explain the cause of their infertility.^[14]^

Currently, the treatment of infertility in western medicine mainly includes reproductive drugs, surgery, or assisted reproductive technology (ART) including in vitro fertilization.^[15–17]^ ART is a new medical application technology. It refers to the in vitro dispose of the human oocyte, sperm or embryo, including all treatments or procedures for initiating a pregnancy.^[18]^ Its main contents consist of in vitro fertilization (IVF), intracytoplasmic sperm injection (ICSI), gamete and embryo cryopreservation, gene diagnosis before embryo implantation, and gene screening before embryo implantation.^[18,19]^ While these treatments have been a boon for infertile patients, the low pregnancy rate, high cost, long cycle, and repeated transplantation failure of IVF technology increase the enormous financial burden and mental pressure on patients.^[20–25]^ These results directly lead to many infertile couples abandoning treatment,^[26]^ or turning to complementary alternative medicine (CAM).^[27–29]^

Acupuncture and moxibustion, as one of important CAM, which has been applied to treating gynecological diseases for thousands of years, has been welcomed by many infertile couples in recent years.^[30–33]^ Moxibustion, as a treatment of acupuncture and moxibustion, refers to the ignition of moxa velvet or stick, then it acts on the corresponding acupoints for burning or fumigation, relying on the role of heat and medicine to achieve the role of disease prevention and treatment. TCM theory holds that moxibustion has the functions of warming and dredging channels and collaterals, regulating qi and blood, dispelling cold and relieving pain, preventing and treating diseases, and strengthening health. It is often used in gynecologic uterine cold caused by infertility, menstrual pain, and metrorrhagia.^[34]^ Modern medical research suggest that moxibustion can improve ovarian function by inhibiting apoptotic events of naturally aging ovaries and enhancing antioxidant defense ability.^[35]^ The stimulation of meridian acupoints by its physical thermo-thermal characteristics and chemical composition of tar from *Artemisia argyi* leaves can activate the self-discipline movement of blood vessels, accelerate blood flow, improve blood circulation, and accelerate local blood circulation.^[36,37]^ Thus, improving ovarian artery blood supply and increasing diastolic blood perfusion can significantly improve ovulation rate and pregnancy rate.^[38]^ Also, animal experiments have found that moxibustion can reduce the expression of p-PI3K, p-Akt, and p-mTOR in rat ovaries. It is suggested that moxibustion may improve ovarian hormone level and inflammatory response by inhibiting the PI3K/Akt/mTOR signaling pathway.^[39]^ Therefore, based on the above experiments, moxibustion has been widely applied to treat female infertility caused by PCOS,^[40]^ premature ovarian failure,^[41]^ tubal obstruction,^[42,43]^ and so on. In recent years, there are more and more studies on the treatment of female infertility by moxibustion. However, to the best of our knowledge, there is no systematic review (SR) at home and abroad to evaluate the efficacy and safety of moxibustion in this field. Therefore, we intend to perform a SR evaluation on the efficacy and safety of moxibustion for female infertility patients through strict review method, hoping to provide a convincing conclusion.

## 2 Methods and analysis

### 2.1 Design and registration of the review

Our SR has been registered on PROSPERO (registration number is CRD42019135593) and the protocol is designed strictly in coordinate with the preferred reporting items of the systematic review and meta-analysis protocol (PRISMA-P).^[44]^ The PRISMA Guidelines and the Cochrane Handbook will be used for the studies we evaluate for inclusion. In addition, bias risk analysis and heterogeneity analysis will also be used in our SR. Subgroup analysis and sensitivity analysis will be further carried out when necessary.

### 2.2 Inclusion criteria

#### 2.2.1 Type of study

We will include randomized controlled clinical trials and quasi-randomized controlled trials. However, studies that used incorrect randomization methods (such as flipping a coin) would not be included. Any other type of literature will be excluded, including moxibustion literature as a non-primary intervention, retrospective research literature, repeated publications, conference abstracts, literature that cannot extract data, case reports, and bibliometric studies. The language limit for searching literature will be limited to Chinese and English due to the language limitation of our researchers.

#### 2.2.2 Types of participants

The study will included women diagnosed with infertility and receiving IVF-ET, ages ranging from 14 to 60, regardless of race, educational level, source of cases, and cause of illness. In addition, repeated IVF failures will also be included. Participants with other serious diseases, such as heart disease, liver disease, kidney disease, or cancer (especially ovarian and breast cancer) will be excluded from the trial.

#### 2.2.3 Types of interventions

The intervention measures should adopt moxibustion alone or moxibustion combined with other methods (exclusion of combination of acupuncture and moxibustion) to treat female infertility, while the control group was treated with non-moxibustion therapy, blank control group, or placebo moxibustion (such as moxa stick not ignited).

#### 2.2.4 Types of outcomes

The main outcomes will be the total effective rate and the clinical pregnancy rate, total effective rate = (total effective number)/total number × 100%, clinical pregnancy rate = (clinical pregnancy number)/total number of pregnancies × 100%.

Secondary outcomes will include the following measures:

(1) Ovulation rate
(2) Endometrial thickness
(3) Hormone level
(4) TCM Syndrome Integral Scale
(5) Adverse event

### 2.3 Data sources and search methods

#### 2.3.1 Electronic searches

We will use computers to search PubMed, Medline, Embase, Web of Science and the Cochrane Central Register of Controlled Trials. Besides, China National Knowledge Infrastructure, China Biomedical Literature Database, China Science Journal Database, and Wan-fang Database will also be collected by our researchers. All databases will be searched from the date of creation to May 31, 2019. The following search terms will be used: infertility, female infertility, polycystic ovary syndrome infertility, premature ovarian failure infertility, tubal infertility, ovulation barrier infertility; moxibustion, moxa leaf, moxa velvet, moxa stick, moxa cone, moxibustion box, ginger-separated moxibustion, dragon moxibustion (Du meridian moxibustion), cake-separated moxibustion, heat-sensitive moxibustion, medicinal moxibustion, sparrow pecking moxibustion, suspension moxibustion; in vitro fertilization, embryo transfer, IVF-ET. The sample search strategy in Table 1 will be used for PubMed. This search strategy will be slightly modified and used in several other databases.

**Table 1:**
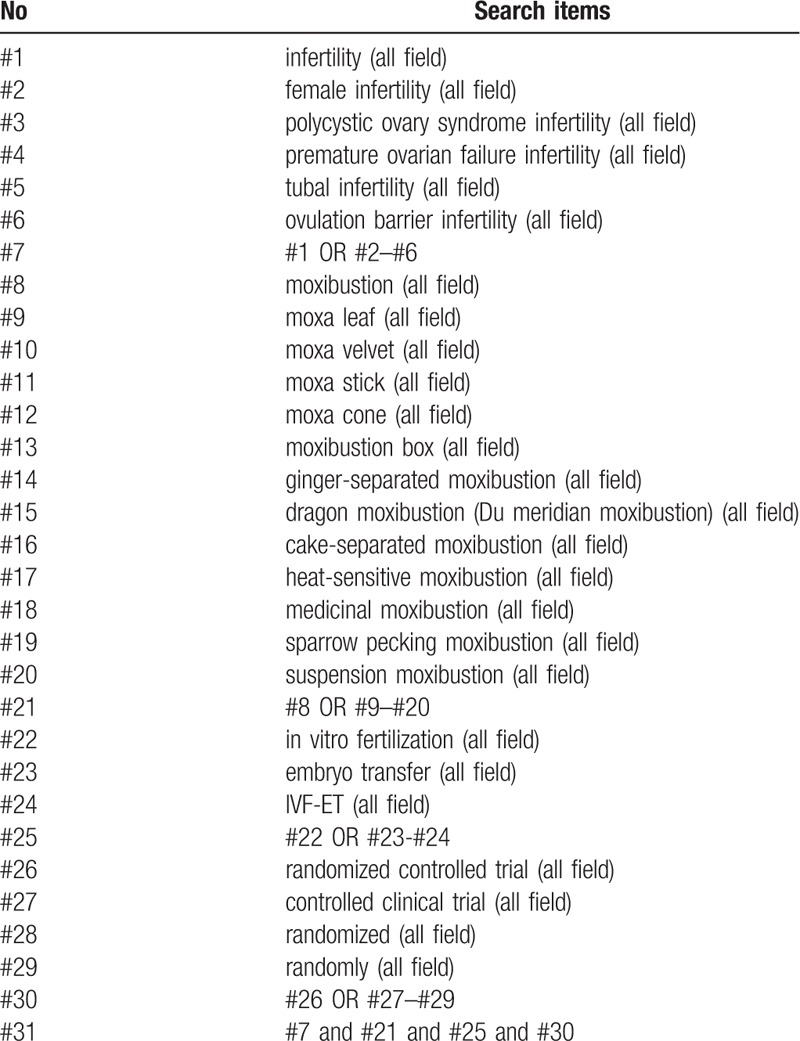
Search strategy used in PubMed.

#### 2.3.2 Searching other resources

For more experiments, we will search for lists of relevant references. We will search the existing SRs of PubMed and Chorane library related to our research topics, and then search their bibliographies for more research. Besides, we will search a reference list to identify published journals, books, conference articles, and gray literature related to the research topic.

### 2.4 Data collection and management

**Figure 1:**
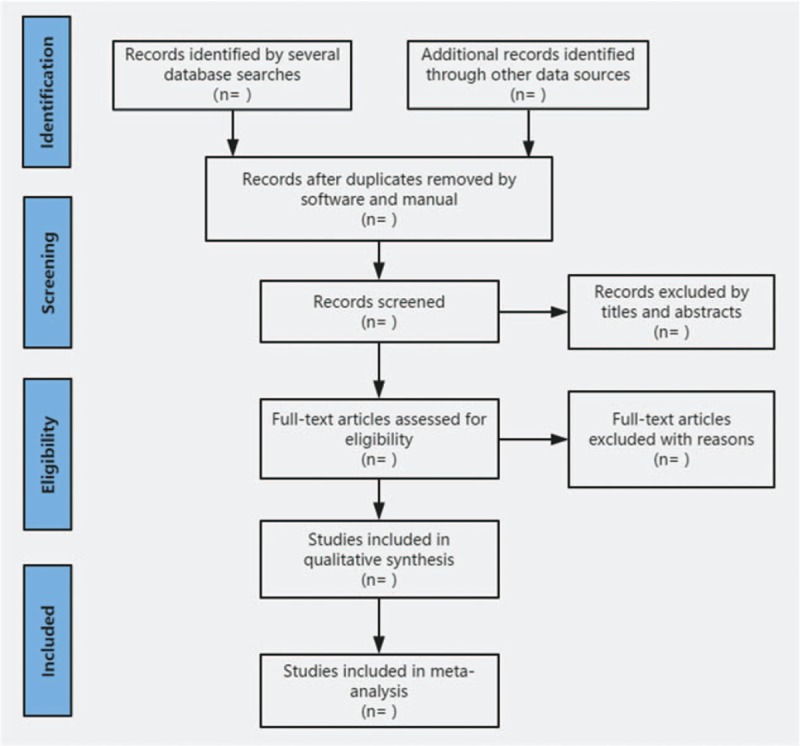
Flow diagram of study selection. This picture reflects the steps of research, selection, and explains the process of literature screening in detail.

After completing all the electronic search work, the result will be imported to Noteexpress software Version 2.6.1 (Aegean Sea software company, Beijing, China) in the same text format, and repeated research will be eliminated by software. Two reviewers (LY and WL) independently complete the screening of documents and then cross-check to determine the final inclusion of documents. In the first stage, all documents after software review will be screened for title, summary, and keywords to determine which documents meet the selection criteria. In the second stage, we will evaluate the full text of the remaining studies and determine whether it meets the SRs criteria. The research excluded after reading the full text will also be documented, and the reasons for exclusion will be recorded. When differences arise in this process, we will invite third parties (ZQH) to arbitrate. The research flow chart is shown in Fig. 1.

### 2.5 Data extraction and analysis

Two researchers (LY and WL) will independently complete data extraction and analysis, and then cross-check the results. In this process, a third author (ZQH) will be invited to resolve any disagreement between the 2 persons. We will produce an Excel spreadsheet to extract literature data, including the first author, country, year of publication, patient characteristics, course of the disease, number of studies, interventions, course of the intervention, outcome indicators, main conclusions, conflicts of interest, recurrence rate, acupoint selection, and adverse events. If the data reported in the document is insufficient, we will contact the author of the experiment for consultation and resolution. However, if we fail to contact the author, the document will be excluded.

### 2.6 Assessment of risk of bias in the included studies

The Cochrane Manual V.5.1.0 tool will be used to assess the risk of bias for each included study. The evaluation includes random sequence generation, allocation sequence hiding, blind evaluation, incomplete result data, selective result report, and other bias sources. The assessment results will be divided into 3 levels: low risk, high risk, and uncertainty risk.

### 2.7 Assessment of heterogeneity

We will test the heterogeneity of data by calculating the value of *I*^2^ statistics and chi-squared test. When *P* > .1, *I*^2^ < 50%, it is considered that there is no great heterogeneity in the study. However, when *P* < .1, *I*^2^ > 50%, it is considered that the study has significant statistical heterogeneity. At this very moment, the subgroup stratification analysis will be further carried out to explore the possible sources of heterogeneity.

### 2.8 Assessment of reporting biases

Funnel chart will be used to assess reporting bias. When the number of studies included exceeds 10 trials, we will use Egger method^[45]^ to test funnel chart asymmetry. If the funnel chart is evenly distributed, it indicates that there is no publication bias.

### 2.9 Data synthesis

We will use RevMan V.5.3 to perform data synthesis. The dichotomous data will be analyzed by relative risk (RR), and the continuous data will be analyzed by the mean difference (MD) or standardized mean difference (SMD). Specific expressions are as follows: when the *I*^2^ test is <50%, the fixed effect model is used to synthesize the data. If the *I*^2^ test is between 50% and 75%, the random effect model is used to synthesize the data. If the *I*^2^ test is >75%, we will conduct a subgroup analysis to analyze possible causes. All data will be analyzed with 95% CIs. If data cannot be synthesized, we will use descriptive analysis to solve this problem.

### 2.10 Subgroup analysis

In the case of high heterogeneity, we will determine the source of heterogeneity by subgroup analysis according to the different combined intervention methods, different treatment courses, different amount of moxibustion, different single moxibustion treatment time, and other different influence factors of female infertility.

### 2.11 Sensitivity analysis

We will conduct a sensitivity analysis to test the recklessness of major decisions in the review process. The main contents of the analysis include the impact of method quality, sample size, and missing data on the study. The meta-analysis will be reused and poor quality research will be excluded. The results will be compared and discussed according to the results.

### 2.12 Grading the quality of evidence

The quality of SRs will be evaluated by using the Grading of Recommendations Assessment, Development, and Evaluation (GRADE).^[46,47]^ Five downgrading factors including risk of bias, inconsistency, indirectness, imprecision, and publication bias will be assessed. The assessment results will be divided into 4 levels: high, moderate, low or very low.

## 3 Discussion

Female infertility is a complex disease with different pathogenic factors. According to WHO report, infertility in the 21st century will become the third-largest disease after cardiovascular, cerebrovascular diseases, and tumors.^[48]^ In addition, the risk of female infertility developing into mental illness is also increasing.^[49]^ At present, many patients and some obstetricians and gynecologists are not satisfied with the treatment results. With the development of CAM, moxibustion, as a traditional method of acupuncture and moxibustion, is widely used in the treatment of gynecological diseases because of its simple operation, long curative effect, low cost, easy acceptance, without needling pain, and no obvious side effects. Nevertheless, there is no English and Chinese publication of SR related to moxibustion for female infertility. This study will collect evidence comprehensively, extract and analyze the data, and draw reasonable and objective conclusions, hoping to provide an evidence-based basis for moxibustion treatment of female infertility and provide more useful information for medical staff and better advice for patients.

## Author contributions

**Conceptualization:** Tinghui Hou.

**Data curation:** Ying Liu, Lu Wang.

**Formal analysis:** Qianhua Zheng.

**Funding acquisition:** Ying Li.

**Investigation:** Tinghui Hou.

**Methodology:** Xiumei Feng.

**Project administration:** Ying Li.

**Supervision:** Qianhua Zheng.

**Writing – original draft:** Tinghui Hou.

**Writing – review and editing:** Qianhua Zheng.
